# Convergent evolution of dogs and humans: a promising new model system for human social behaviors and mental health

**DOI:** 10.1093/nsr/nwag405

**Published:** 2026-07-02

**Authors:** Yan-Hu Liu, Yun Yu, Enikő Kubinyi, Ya-Ping Zhang

**Affiliations:** State Key Laboratory of Genetic Evolution & Animal Models and Yunnan Key Laboratory of Molecular Biology of Domestic Animals, Kunming Institute of Zoology, Chinese Academy of Sciences, China; KIZ-CUHK Joint Laboratory of Bioresources and Molecular Research in Common Diseases, Kunming Institute of Zoology, Chinese Academy of Sciences, China; State Key Laboratory of Genetic Evolution & Animal Models and Yunnan Key Laboratory of Molecular Biology of Domestic Animals, Kunming Institute of Zoology, Chinese Academy of Sciences, China; KIZ-CUHK Joint Laboratory of Bioresources and Molecular Research in Common Diseases, Kunming Institute of Zoology, Chinese Academy of Sciences, China; Department of Ethology, ELTE Eötvös Loránd University, Hungary; State Key Laboratory of Genetic Evolution & Animal Models and Yunnan Key Laboratory of Molecular Biology of Domestic Animals, Kunming Institute of Zoology, Chinese Academy of Sciences, China; KIZ-CUHK Joint Laboratory of Bioresources and Molecular Research in Common Diseases, Kunming Institute of Zoology, Chinese Academy of Sciences, China; Kunming College of Life Science, University of Chinese Academy of Sciences, China; Bio-X Center for Interdisciplinary Innovation, Yunnan University, China

Dogs (*Canis lupus familiaris*) were domesticated from an extinct gray wolf lineage at least 15 000 years ago [1–5]. Strong artificial selection has produced over 450 recognized breeds with remarkably diverse traits, most of which were established within the last 200 years. As humans’ closest companion animals, dogs uniquely combine shared living environments and resources with multifaceted societal roles in work and service. Driven by their deeply intertwined ecological niche and convergent evolution, dogs and humans have evolved striking similarities in social behavior and cognition, resulting in exceptional interspecific sociability and communication. These characteristics make dogs a promising new large-animal model for studying mechanisms underlying social behaviors and mental disorders.

**Convergent evolution of dogs and humans in social behaviors.** Domestication not only reshaped the morphology and behavior of animals, but also facilitated the transition from hunter-gatherer societies to permanent or semipermanent settlements. In 1868, Darwin described the ‘domestication syndrome’ revealing that domesticated animals possess behavioral, physiological and morphological traits absent in their wild ancestors [6]. He also noted that humans display similar features, a concept that progressively developed into the human self-domestication hypothesis (Table S1), which proposes that selection for reduced aggression and enhanced prosociality may have driven analogous phenotypic changes in humans [7] (Fig. 1A).

**Figure 1.**
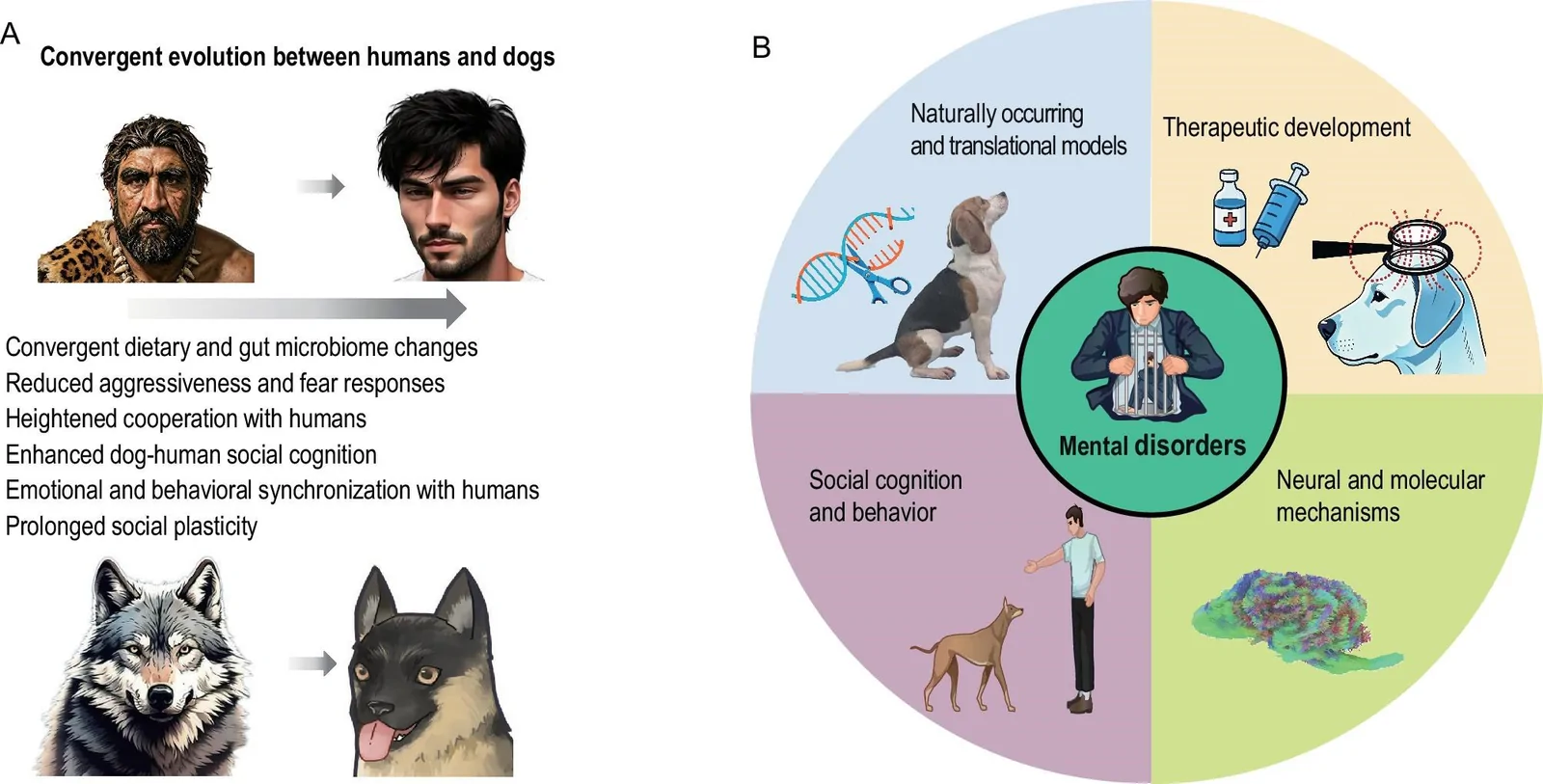
Dogs as potential models for studying human social behaviors and mental health. (A) Human evolution has led to a series of changes that parallel those observed in domesticated dogs. (B) Overview of current research aims using dogs as models.

Most domesticated animals were bred primarily for production traits [8]. In contrast, dogs were initially selected for behaviors and later for aesthetics, leading to remarkable social skills and specialized adaptations that make them well-suited for human societies during and after the Agricultural Revolution. Csányi proposed that the three core domains of human behavior complex (sociality, synchronization and constructive ability) changed during human social evolution, with dogs exhibiting functionally analogous traits to humans in these domains [9,10]. We briefly outline this behavioral framework in Note S1, and further discuss their potential genetic basis in Note S2.

**Convergent evolution in diet and gut microbiomes between dogs and humans.** The Agricultural Revolution not only catalyzed increasingly complex societal structures and behaviors, but also profoundly altered the diets and digestive systems of dogs and humans. Crucially, this shared dietary shift has driven convergent evolution of metabolic functions within the gut microbiome [11]. Coupled with evidence linking the gut microbiome to cognition, stress resilience and social behavior, this convergence suggests that dog–human microbiome interactions may provide new insights into the biological underpinnings of mental health [12]. Together with convergent social and cognitive traits, these microbiome similarities further support the value of dogs as comparative models for investigating the biological basis of behavior and mental health.

**Striking similarities in mental disorders between dogs and humans.** Among domesticated species, dogs exhibit one of the largest numbers of characterized hereditary diseases. The Online Mendelian Inheritance in Animals database currently records 1010 canine phenes, including 647 phenes resembling human diseases, making dogs valuable natural models for identifying candidate variants and pathogenic mechanisms for both species (https://omia.org/home/, accessed 29 May 2026) [13]. For example, an enhancer associated with canine separation anxiety modulates *SLC32A1*, a gene also linked to schizophrenia and depression in humans [14]. Although numerous candidate genes have been associated with canine diseases, only a limited number of underlying mechanisms have been validated. Nevertheless, hereditary diseases in dogs offer a unique comparative framework for uncovering shared and divergent aspects of genetics, etiology and disease mechanisms in both species.

**Dogs as a model to understand human social behaviors.** Dogs exhibit extreme behavioral diversity and close integration with human societies. Their high inter-breed diversity and low intra-breed genetic diversity provide an excellent system for investigating the genetic basis of social traits. While current behavioral studies on dogs are dominated by purebreds with limited work on free-ranging dogs, indigenous dogs, which are integrated into human households and have never experienced breeding. Future research should prioritize four directions: (i) comparing social interactions with conspecifics and humans across free-ranging dogs (self-sustaining), purebred dogs (extreme artificial selection), indigenous dogs (early stages of dog domestication) and wolves (ancestral); (ii) identifying key genes and regulatory networks involved in dog domestication and behavioral diversity, and in parallel, in human evolution; (iii) investigating the function of candidate genes at multiple levels: cells, tissues and individuals; and (iv) investigating how evolutionary adaptations and modern social conditions interact to influence vulnerability to mental disorders.

**Dogs as a promising new model for studying mental disorders.** Mental disorders are a global health crisis, and traditional rodent models show limited translational success [15]. Dogs share similarities with humans in brain structure and function, behavior complexity and gene expression across tissues. Additionally, dogs with spontaneous severe behavioral abnormalities are potential models for psychiatric disorders. Thus, dogs provide a promising new large-animal model for studying mental disorders. Under rigorous welfare frameworks, dog model characterization should integrate behavioral assessments, neuroimaging and multi-omics data to link behaviors to neural function and molecular pathways (Fig. 1B). To ensure translational relevance, these canine-derived findings require close comparison with human clinical data. Ultimately, such integrative approaches can guide the development of chemical and non-invasive interventions for social impairments in humans and dogs. However, current behavioral quantification relies heavily on manual scoring, which is labor-intensive and prone to subjective bias. Novel artificial intelligence (AI)-based approaches are emerging to accurately and efficiently quantify behaviors and generate behavioral atlases. In parallel, dog-specific equipment for monitoring biological signals related to social behavior should be developed and standardized.

**Balancing scientific necessity and animal welfare in dog psychiatric models.** Dog model studies require particularly careful ethical consideration because of their advanced social-cognitive abilities, close relationships with humans and special status in society. Consequently, developing canine models for social impairments demands responsible, welfare-centered approaches. Crucially, dog models should prioritize dogs with naturally occurring behavioral abnormalities. Gene-editing strategies should be considered only as a secondary option when spontaneous models are unavailable and restricted exclusively to laboratory-bred beagles following stringent scientific justification. Candidate genes should first be validated in cellular and rodent models prior to canine application. All studies must place animal welfare at the forefront, undergo rigorous ethical review and maintain heightened sensitivity to societal expectations. In strict adherence to the Principle of Reduction, comprehensive data and biological samples must be maximally leveraged from each individual dog to minimize the total number of animals required.

In conclusion, dogs provide unique opportunities to investigate convergent evolution in behavioral and psychiatric traits. Their exceptional sociability, rich behavioral diversity and advanced genetic tools bridge evolutionary biology with neuroscience and psychiatry. Applying advances in genomics, neurobiology and AI to those models enables detailed investigation of the mechanisms underlying social behavior and mental health. By aligning scientific advances with responsibility and transparency, these studies not only inform human mental health but also support dog welfare and the broader One Health agenda.

## Supplementary Material

nwag405_Supplemental_File
