# Supplementary material for: Convergent evolution of dogs and humans: a promising new model system for human social behaviors and mental health
Source: Natl Sci Rev. 2026 Jul 2;13(20):nwag405. doi: 10.1093/nsr/nwag405 (PMC13725830; doi:10.1093/nsr/nwag405)
Supplement: Supplementary file 1 [file nwag405_supplemental_file.docx]

Supplementary Note 1: A functional comparison of the behavioral complex between dogs and humans based on Csányi’s framework.

1) **Sociality**: behaviors related to group life (e.g., social attraction, food sharing, decreased intergroup aggression, complementary cooperation). Due to their long-term companionship with humans, dogs have adapted to and become integrated into human social environments. Many components of dog behavior are enhanced and functionally analogous to those of humans, while their intraspecific social skills remain unchanged or may even have declined [1]. For example, dogs have evolved specific adaptations in their facial muscle anatomy to facilitate communication with humans [2]. Compared to wolves, dogs generally display lower levels of intragroup aggression and enhanced cooperation with humans (Supplementary Table S1); however, it remains debated whether they truly exhibit less aggression than wolves, and whether this “cooperation” reflects evolved cooperative abilities, learned responsiveness to humans, or both [3-6]. Moreover, dogs show a strong inclination to form lasting attachment bonds with humans, often developing them rapidly and maintaining them throughout their lives.

2) **Synchronization**: mechanisms that facilitate coordinated group activity (e.g., behavioral and emotional alignment, imitation, and rule-following). Dogs exhibit pronounced abilities in behavioral and emotional synchronization with humans, as well as in imitation and compliance with social rules [6]. Moreover, evidence for interbrain synchronization has recently been reported in interacting human–dog dyads [7], highlighting the neuronal basis of this capacity. These abilities may parallel mechanisms of group cohesion in humans and may reflect convergent adaptations that support complex social living.

3) **Constructive abilities**: the brain’s capacity to reduce situations to their components and construct new structures, including forms of abstraction, symbolic learning, and flexible problem solving. Domestication has enhanced dogs’ sensitivity to specific human cues like pointing and gaze [8]. Through vocalizations, eye contact, body gestures, and sensitivity to human emotions (Fig. 1B), dogs engage in effective interspecies communication and cooperation. Although observed in limited samples, dogs have demonstrated behavioral innovation when prompted by a “create” command [9]. Moreover, Gifted Word Learner dogs not only extend known verbal markers to functionally equivalent items [10], but also acquire new labels simply by eavesdropping on human interactions [11]. These findings suggest that certain dogs exhibit cognitive flexibility extending beyond simple associative learning.

Supplementary Note 2: Genetic convergent evolution between dog domestication and human

self-domestication.

Comparative genomic studies have revealed that a series of genes under positive selection in both humans and domesticated animals are involved in neurological processes [12, 13] and neural crest pathways [14-18]. Neural crest cells arise from the dorsal edge of the neural tube and migrate ventrally throughout the embryo to form various tissues. The neural crest hypothesis suggests that mutations in genes affecting neural crest cell activity during embryonic development are central to the "domestication syndrome" [19]. If validated, this framework could help explain the parallel behavioral and morphological traits observed in both dog domestication and human self-domestication. Although compelling, this hypothesis requires more rigorous testing, including comparative genomics across diverse species, reconstruction of regulatory networks from multi-omics data, and functional experimental evidence. While improved diagnostics and healthcare access contribute to higher detection rates of mental disorders, some scientists argue that mismatches between genetic adaptations and modern environments contribute to the increasing prevalence of mental disorders in humans [20, 21].

Table S1 Same phenotypic changes between dog domestication and human self-domestication

| Traits | References for humans | References for dogs |
| --- | --- | --- |
| Reduced aggressiveness and fear | [22, 23] | [24-33]; But it may be due to the socio-ecological niche changes: dogs rely less on cooperative hunting than wolves [3, 34]. |
| Increased social tolerance and prosociality | [35, 36] | [37, 38] |
| Increased cooperative behavior | [39] | [1, 8, 38, 40-43]; But it is possible that dogs treat humans as a leader and obey him, rather than see human as a cooperator [3, 5]. |
| Expanded neoteny and juvenilization | [44-46] | [38, 47] |
| Salient craniofacial differences | [35] | [2, 15] |
| Smaller teeth | [48] | [24] |
| Smaller cranial capacity | [49] | [50, 51] |
| Altered serotonergic system | [52] | [12, 53, 54] |
| Altered oxytocin system | [55] | [29, 56-59] |

References

1.	Ferreira VHB, Lansade L, Calandreau L *et al.* Are domesticated animals dumber than their wild relatives? A comprehensive review on the domestication effects on animal cognitive performance. *Neuroscience & Biobehavioral Reviews*. 2023; **154**: 105407.

2.	Kaminski J, Waller BM, Diogo R *et al.* Evolution of facial muscle anatomy in dogs. *Proc Natl Acad Sci U S A*. 2019; **116**(29): 14677–14681.

3.	Range F, Marshall-Pescini S. Comparing wolves and dogs: current status and implications for human ‘self-domestication’. *Trends Cogn Sci*. 2022; **26**(4): 337–349.

4.	Kubinyi E, Gácsi M, Topál J *et al.* Dog–wolf differences: caution is needed to avoid overgeneralisation of scanty data. *Trends Cogn Sci*. 2022; **26**(9): 728–729.

5.	Range F, Marshall-Pescini S. Taking confounding factors and life experience seriously. *Trends Cogn Sci*. 2022; **26**(9): 730–731.

6.	Gácsi M. After all, how valuable is the dog model for examining human socio-cognitive abilities? *Biologia Futura*. 2025; **76**: 193–212.

7.	Ren W, Yu S, Guo K *et al.* Disrupted Human–Dog Interbrain Neural Coupling in Autism-Associated Shank3 Mutant Dogs. *Advanced Science*. 2024; **11**(41): 2402493.

8.	Hare B, Brown M, Williamson C *et al.* The domestication of social cognition in dogs. *Science*. 2002; **298**(5598): 1634–1636.

9.	Scagel A, Hege AS, III EM. Exploring Dogs’Capacity to Innovate Actions on Cue. *Animal Behavior and Cognition*. 2024; **11**(2): 167–197.

10.	Fugazza C, Sommese A, Miklósi Á. Dogs extend verbal labels for functional classification of objects. *Curr Biol*. 2025; **35**(19): 4820–4826.e4823.

11.	Dror S, Miklósi Á, Morvai B *et al.* Dogs with a large vocabulary of object labels learn new labels by overhearing like 1.5-year-old infants. *Science*. 2026; **391**(6781): 160–163.

12.	Wang GD, Zhai W, Yang H-c *et al.* The genomics of selection in dogs and the parallel evolution between dogs and humans. *Nat Commun*. 2013; **4**: 1860.

13.	Wang GD, Zhai W, Yang H-C *et al.* Out of southern East Asia: the natural history of domestic dogs across the world. *Cell Res*. 2016; **26**(1): 21–33.

14.	Pendleton AL, Shen F, Taravella AM *et al.* Comparison of village dog and wolf genomes highlights the role of the neural crest in dog domestication. *BMC Biol*. 2018; **16**(1): 64.

15.	Theofanopoulou C, Gastaldon S, O’Rourke T *et al.* Self-domestication in *Homo sapiens*: Insights from comparative genomics. *PLoS One*. 2017; **12**(10): e0185306.

16.	Rubio AO, Summers K. Neural crest cell genes and the domestication syndrome: A comparative analysis of selection. *PLoS One*. 2022; **17**(2): e0263830.

17.	Šimić G, Vukić V, Kopić J *et al.* Molecules, Mechanisms, and Disorders of Self-Domestication: Keys for Understanding Emotional and Social Communication from an Evolutionary Perspective. *Biomolecules*. 2021; **11**(1): 2.

18.	Zanella M, Vitriolo A, Andirko A *et al.* Dosage analysis of the 7q11.23 Williams region identifies *BAZ1B* as a major human gene patterning the modern human face and underlying self-domestication. *Sci Adv*. 2019; **5**(12): eaaw7908.

19.	Wilkins AS, Wrangham RW, Fitch WT. The “domestication syndrome” in mammals: a unified explanation based on neural crest cell behavior and genetics. *Genetics*. 2014; **197**(3): 795–808.

20.	Bednarik RG, Saniotis A, Henneberg M. Auto-domestication hypothesis and the rise in mental disorders in modern humans. *Medical Hypotheses*. 2022; **164**: 110874.

21.	Koller D, Benítez-Burraco A, Polimanti R. Enrichment of self-domestication and neural crest function loci in the heritability of neurodevelopmental disorders. *Hum Genet*. 2023; **142**(8): 1271–1279.

22.	Wrangham R, Pilbeam D. African apes as time machines. *All Apes Great and Small: Volume 1: African Apes*: Springer; 2002. 5–17.

23.	Hare B, Wobber V, Wrangham R. The self-domestication hypothesis: evolution of bonobo psychology is due to selection against aggression. *Anim Behav*. 2012; **83**(3): 573–585.

24.	Darwin C. *The variation of animals and plants under domestication*. New York: Authorized ed. O. Judd & company, 1868.

25.	Belyaev DK. Domestication of animals. *Science*. 1969; **5**(1): 47–52.

26.	Boitani L, Ciucci P. Comparative social ecology of feral dogs and wolves. *Ethol Ecol Evol*. 1995; **7**(1): 49–72.

27.	Trut LN, Plyusnina IZ, Oskina IN. An experiment on fox domestication and debatable Issues of evolution of the dog. *RUSS J GENET*. 2004; **40**(6): 644–655.

28.	MacDonald DW, Carr GM. Variation in dog society: Between resource dispersion and social fl ux. In: Serpell J (ed.) *The Domestic Dog: Its Evolution, Behavior and Interactions with People*. Cambridge: Cambridge University Press; 2016. 319–341.

29.	Wang GD, Xie HB, Peng MS *et al.* Domestication genomics: evidence from animals. *Annu Rev Anim Biosci*. 2014; **2**(1): 65–84.

30.	Gácsi M, Győri B, Miklósi Á *et al.* Species-specific differences and similarities in the behavior of hand-raised dog and wolf pups in social situations with humans. *Dev Psychobiol*. 2005; **47**(2): 111–122.

31.	Pal SK. Factors influencing intergroup agonistic behaviour in free-ranging domestic dogs (Canis familiaris). *acta ethologica*. 2015; **18**(2): 209–220.

32.	Wirobski G, Range F, Graat EAM *et al.* Similar behavioral but different endocrine responses to conspecific interactions in hand-raised wolves and dogs. *iScience*. 2023; **26**(2): 105978.

33.	Cafazzo S, Marshall-Pescini S, Lazzaroni M *et al.* The effect of domestication on post-conflict management: wolves reconcile while dogs avoid each other. *R Soc Open Sci*. 2018; **5**(7): 171553.

34.	Marshall-Pescini S, Cafazzo S, Virányi Z *et al.* Integrating social ecology in explanations of wolf–dog behavioral differences. *Current Opinion in Behavioral Sciences*. 2017; **16**: 80–86.

35.	Cieri RL, Churchill SE, Franciscus RG *et al.* Craniofacial feminization, social tolerance, and the origins of behavioral modernity. *Curr Anthropol*. 2014; **55**(4): 419–443.

36.	Henrich J. *The Secret of Our Success: How Culture Is Driving Human Evolution, Domesticating Our Species, and Making Us Smarter*. Princeton: Princeton University Press, 2018.

37.	Hemmer H. *Domestication: the decline of environmental appreciation*: Cambridge: Cambridge University Press, 1990.

38.	Miklósi Á. *Dog Behaviour, Evolution, and Cognition*: Oxford University Press, 2014.

39.	Boyd R, Richerson PJ. Cultural transmission and the evolution of cooperative behavior. *Hum Ecol*. 1982; **10**(3): 325–351.

40.	Hare B, Tomasello M. Human-like social skills in dogs? *Trends Cogn Sci*. 2005; **9**(9): 439–444.

41.	Miklósi Á, Kubinyi E, Topál J *et al.* A simple reason for a big difference: wolves do not look back at humans, but dogs do. *Curr Biol*. 2003; **13**(9): 763–766.

42.	Gácsi M, Győri B, Virányi Z *et al.* Explaining dog wolf differences in utilizing human pointing gestures: selection for synergistic shifts in the development of some social skills. *PLoS One*. 2009; **4**(8): e6584.

43.	Topál J, Gergely G, Erdőhegyi Á *et al.* Differential sensitivity to human communication in dogs, wolves, and human infants. *Science*. 2009; **325**(5945): 1269–1272.

44.	Shea BT. Heterochrony in human evolution: The case for neoteny reconsidered. *Am J Phys Anthropol*. 1989; **32**(S10): 69–101.

45.	Meng X, Lin Q, Zeng X *et al.* Brain developmental and cortical connectivity changes in transgenic monkeys carrying the human-specific duplicated gene *SRGAP2C*. *Natl Sci Rev*. 2023; **10**(11): nwad281.

46.	Wang L, Pang K, Zhou L *et al.* A cross-species proteomic map reveals neoteny of human synapse development. *Nature*. 2023; **622**(7981): 112–119.

47.	Fox MW. The anatomy of aggression and its ritualization in Canidae: a developmental and comparative study. *Behaviour*. 1969: 242–258.

48.	Kupczik K, Hublin J-J. Mandibular molar root morphology in Neanderthals and Late Pleistocene and recent *Homo sapiens*. *J Hum Evol*. 2010; **59**(5): 525–541.

49.	Ponce de León MS, Golovanova L, Doronichev V *et al.* Neanderthal brain size at birth provides insights into the evolution of human life history. *Proc Natl Acad Sci U S A*. 2008; **105**(37): 13764–13768.

50.	Kruska D. Mammalian Domestication and its Effect on Brain Structure and Behavior. In: *Berlin, Heidelberg,* *1988*, p. 211–250. Springer Berlin Heidelberg.

51.	Garamszegi LZ, Kubinyi E, Czeibert K *et al.* Evolution of relative brain size in dogs—no effects of selection for breed function, litter size, or longevity. *Evolution*. 2023; **77**(7): 1591–1606.

52.	Lew CH, Hanson KL, Groeniger KM *et al.* Serotonergic innervation of the human amygdala and evolutionary implications. *Am J Phys Anthropol*. 2019; **170**(3): 351–360.

53.	Kikusui T, Nagasawa M, Nomoto K *et al.* Endocrine regulations in human-dog coexistence through domestication. *Trends Endocrinol Metab*. 2019; **30**(11): 793–806.

54.	Li Y, Wang G-D, Wang M-S *et al.* Domestication of the dog from the wolf was promoted by enhanced excitatory synaptic plasticity: a hypothesis. *Genome Biol Evol*. 2014; **6**(11): 3115–3121.

55.	Schaschl H, Huber S, Schaefer K *et al.* Signatures of positive selection in the cis-regulatory sequences of the human oxytocin receptor (*OXTR*) and arginine vasopressin receptor 1a (*AVPR1A*) genes. *BMC Evol Biol*. 2015; **15**: 85.

56.	Wirobski G, Range F, Schaebs FS *et al.* Endocrine changes related to dog domestication: Comparing urinary cortisol and oxytocin in hand-raised, pack-living dogs and wolves. *Horm Behav*. 2021; **128**: 104901.

57.	Kis A, Bence M, Lakatos G *et al.* Oxytocin receptor gene polymorphisms are associated with human directed social behavior in dogs (*Canis familiaris*). *PLoS One*. 2014; **9**(1): e83993.

58.	Oliva JL, Wong YT, Rault J-L *et al.* The oxytocin receptor gene, an integral piece of the evolution of *Canis familaris* from *Canis lupus*. *Pet Behav Sci*. 2016; **2**: 1–15.

59.	Bence M, Marx P, Szantai E *et al.* Lessons from the canine *Oxtr* gene: populations, variants and functional aspects. *Genes Brain Behav*. 2017; **16**(4): 427–438.
